# Interventions to Improve Adherence to Antiretroviral Therapy (ART) in Sub-Saharan Africa: An Updated Systematic Review

**DOI:** 10.3390/ijerph18052477

**Published:** 2021-03-03

**Authors:** Panmial Priscilla Damulak, Suriani Ismail, Rosliza Abdul Manaf, Salmiah Mohd Said, Oche Agbaji

**Affiliations:** 1 Department of Community Health, Faculty of Medicine and Health Sciences, Universiti Putra Malaysia, Seri Kembangan 43400, Malaysia; damulakpanmial@yahoo.com (P.P.D.); rosliza_abmanaf@upm.edu.my (R.A.M.); salmiahms@upm.edu.my (S.M.S.); 2 Department of Public Health, Faculty of Basic Medical Sciences, Baze University, Abuja, Plot 686, Cadastral Zone C 00, Nigeria; 3 AIDS Prevention Initiative in Nigeria, Jos University Teaching Hospital, Jos 2076, Nigeria; agbajio@unijos.edu.ng

**Keywords:** interventions, adherence, antiretroviral therapy, sub-Saharan Africa

## Abstract

Optimal adherence to antiretroviral therapy (ART) remains the bedrock of effective therapy and management of human immunodeficiency virus (HIV). This systematic review examines the effect of interventions in improving ART adherence in sub-Saharan Africa (SSA), which bears the largest global burden of HIV infection. In accordance with PRISMA guidelines, and based on our inclusion and exclusion criteria, PUBMED, MEDLINE, and Google Scholar databases were searched for published studies on ART adherence interventions from 2010 to 2019. Thirty-one eligible studies published between 2010 to 2019 were identified, the categories of interventions were structural, behavioral, biological, cognitive, and combination. Study characteristics varied across design, intervention type, intervention setting, country, and outcome measurements. Many of the studies were behavioral interventions conducted in hospitals with more studies being randomized controlled trial (RCT) interventions. Despite the study variations, twenty-four studies recorded improvements. Notwithstanding, more quality studies such as RCTs should be conducted, especially among key affected populations (KAPs) to control transmission of resistant strains of the virus. Reliable objective measures of adherence should replace the conventional subjective self-report. Furthermore, long-term interventions with longer duration should be considered when evaluating the effectiveness of interventions.

## 1. Introduction

Since highly active antiretroviral therapy (HAART) is the standard treatment for HIV-positive patients, the effectiveness of antiretroviral therapy (ART) varies majorly with patient’s adherence observance to the daily medication regimen. One of the major concerns of public health for people living with HIV (PLWHIV) is the promotion of medication adherence [1]. Although structural, social, and personal factors could be reasons for failure to adhere to ART among patients, [2,3] other factors such as health-system-related barriers, food insecurity, supply-chain interruptions, and insufficient human health resources, are barriers to ART adherence in Africa [4]. Through the years, adherence has been found to be a fundamental predictor of ART treatment success acquiescent to intervention [2] however, several patients are found to lapse on the prescribed treatment regimen thereby increasing the risk of transmitting HIV, deteriorating health conditions [5], therapy failure, production of new resistant viral strains, progression to acquired immune deficiency syndrome (AIDS), more hospitalization and increased rates of mortality [6,7,8], and poor quality of life. Consequently, the resultant effect of not adhering to ART is increased cost of healthcare. Unfortunately, two-thirds of PLWHIV are found in developing countries, particularly sub-Saharan Africa (SSA), most of who are economically disadvantaged [9].

Optimal ART adherence is fundamental to achieving HIV viral suppression and improving well-being of HIV-positive patients. Other benefits of ART adherence include decline in morbidity and mortality rates, decreased probability of transmitting the virus to sero-negative partners, and improved quality of life [10,11,12,13]. The effectiveness of ART, and even among those diagnosed and placed on therapy is reflected in the 18% surge in viral suppression among all HIV-positive patients globally between 2015 and 2019. However in 2019, only 59% of HIV-positive patients had suppressed viral loads, which indicated the unfeasibility of achieving the 90-90-90 target of 2020 [14]. The 90-90-90 target is a United Nations declaration to bring AIDS to an end by HIV testing, treatment, and viral suppression. Being the goal of HIV treatment, viral suppression to undetectable levels is a key strategy to ending the pandemic.

A global snowballing trend in HIV prevalence and significant downswing in AIDS-related deaths is suggestive of the existing gains of ART. Since sub-Saharan Africa has the bulk of global disease burden of HIV (70%), success in prevention of the disease would have an impact on the global disease burden [15]. Despite efforts and extensive advancement in ART scale up exercises, 1.7 million people were infected globally with about two-thirds (1.1 million) recorded from Africa in 2018. In the same year, 770,000 AIDS-related deaths were recorded globally, of which 470,000 were in the African Region [16]. While the gains of treatment are recognized, poor adherence builds a gap between prospective and accomplished public health rewards of ART [17].

Several studies have been carried out to improve adherence to ART using different interventions with varying outcomes. A systematic review was conducted in 2011 to assess evidence of the effect of interventions on ART adherence in SSA [18]. The result revealed that diary cards, directly observed therapy, food rations, treatment supporters, and cell phone short message services effectively improve adherence in SSA; although some interventions were reported to produce an ephemeral effect, others were not effective in all settings. These findings suggest that more research is required, particularly RCTs, which examine the specific content on effectiveness of interventions [19]. However, it should be notably stated that data from interventions on adherence conducted in developed countries might be ineffective or have less relevance in African setting. This could be due to contextual differences and distinctive characteristics ranging from healthcare personnel involved in service delivery to healthcare access. Interventions in SSA are tailored to specific needs of the populations involved (such as female sex workers, orphans, and widows) or location (rural or urban and hospital-based or home-based). Interventions that are capital or resource-intensive may unlikely be conducted in SSA due to limited resources [18].

So, this systematic review updates the findings of the previous systematic review [18] on effectiveness of ART adherence interventions in SSA, by reporting latest evidence-based interventions conducted in this region of Africa. It also seeks to identify newer strategies of improving adherence interventions.

## 2. Materials and Methods

This systematic review used the preferred reporting items for systematic review and meta-analysis (PRISMA) statement guidelines of 2009 [19]. The PRISMA guidelines enable authors to improve the reporting of protocols for intended systematic reviews and meta-analyses, by providing them with minimum requirements for a protocol. It is an evidence-based minimum set of items for reporting in systematic reviews and meta-analyses. Due to the aggregate nature of the study no informed consents or IRB approval were required.

### 2.1. Search Strategy

The literature search included PUBMED, MEDLINE, and Google Scholar databases, published studies from 2010 to 2019 with the aid of some selected terms in titles and abstracts. The search was achieved using the Boolean operator “and” and “or”. The key search words combining the medical subject headings (MeSHs) “interventions” or “strategies”, and “Antiretroviral Therapy” or “highly active antiretroviral therapy” or “antiretroviral” or “anti-HIV agents” or “ART” or “ARV” and “adherence” or “compliance” and “Africa” or “sub-Saharan”. A manual search was also done on Google Scholar to explore the grey literature as well. The reference lists of articles from these journals were also searched; this was done by searching the terms “adherence”, “antiretroviral therapy”, “antiretroviral”, “ART”, and “intervention”. The literature search was done by P.P.D. and S.I. Studies were screened by two reviewers (P.P.D and S.I) independently while disparities were resolved by R.A.M., S.M.S., and A.O. The reporting of the findings of this systematic review are in line with the PRISMA guidelines.

### 2.2. Eligibility Criteria

This review was updated from a previous review [18] that involved studies evaluating interventions’ effectiveness to improve adherence to ART in adults in sub-Saharan Africa with adherence as the primary or secondary outcome. The definition of adherence in this review was operationally restricted to ART adherence, which implied the degree of medication (antiretroviral) intake by patients as recommended by their providers of healthcare. Studies relating to the distinct concepts of adherence such as clinic attendance or appointments and retention were equally reviewed. There was no restriction on the measures of assessment of ART adherence. The guide used for the inclusion criteria was the population, intervention, comparison, outcome, and time (PICOT) mnemonics [19,20]. 

Population: All adult HIV-positive patients on ART. Studies involving only children were excluded. 

Intervention: Interventions to improve ART adherence and biological correlates of adherence. 

Comparison: Studies with a comparison group or control group were included. However, studies with no direct comparison group, for instance, some quasi experimental studies were excluded from the study.

Outcome: Adherence to ART and correlates of adherence.

Time: Studies published from 2010 to 2019 were included.

There were no exclusion criteria for study designs. Unpublished trials were not included and only journal articles published in the English language were reviewed. The review contained only studies from SSA, and only studies involving SSA sites were included for multisite studies. According to the exclusion criteria, studies that were excluded include studies that were not journal articles, which involved only children, not reporting any adherence intervention, not involving a comparison group or control, and not reporting adherence-related outcomes. Studies that mentioned adherence in their titles but did not actually measure adherence were excluded.

### 2.3. Study Selection

Studies were reviewed based on strict adherence to ART appointments and medication as scheduled by their health care providers. By sequence, articles were screened according to title, abstract, and full text to ascertain their inclusion. Studies involving interventions relating to ART adherence in sub-Saharan Africa were included in the review, which reported adherence measurements conducted alongside interventions. From each article that passes the screening above, information on year of publication, type of intervention, country where study was conducted, health care setting, and outcomes were reported. Subjective and objective measures of adherence were recorded, including biological correlates of adherence, for instance, viral load and CD4 count. A total of thirty-one studies were included in the final analysis, and these studies were screened by two independent reviewers (P.P.D and S.I) while R.A.M, S.M.S, and A.O reviewed the selection and resolved disagreements.

### 2.4. Quality Assessment

The Cochrane criteria were used for the systematic assessment of bias for the studies included in this review. The risk of bias was evaluated as either “low risk”, “high risk”, or “unclear risk” analyzed over seven domains [21,22,23]. Low risk indicates reported information with evidence of little or no possible bias while high risk implies evidence of possible bias. Unclear risk denotes a dearth of info or skepticism over possible bias. Among the domains were sequence generation, allocation sequence, concealment, blinding (participants and personnel), blinding of outcome assessment, incomplete outcome data, selective outcome reporting, and other potential sources of bias. The opinion of a third reviewer was sought to solve disagreements. 

## 3. Results

**Figure 1:**
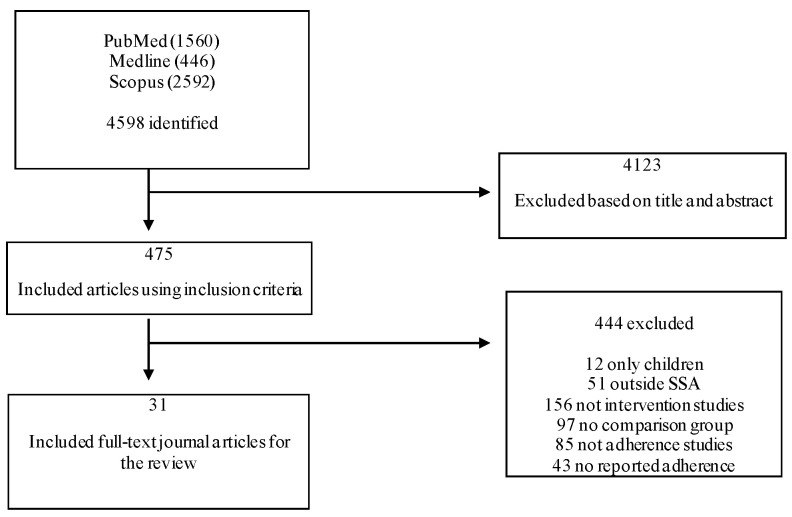
Flowchart of the systematic review according to PRISMA guidelines 2009.

A total of 4598 records (1560 in PubMed, 446 in Medline, and 2592 in Scopus) were identified, of which 4123 were excluded based on the content of their titles and abstracts, which was not in line with the inclusion criteria. Studies that did not measure adherence as an outcome were excluded from the systematic review. Thirty-one journal articles met the inclusion criteria [24,25,26,27,28,29,30,31,32,33,34,35,36,37,38,39,40,41,42,43,44,45,46,47,48,49,50,51,52,53,54] and were included in the systematic review. Figure 1 shows the flowchart of the systematic review process.

### 3.1. Study Characteristics

Table 1 presents the study characteristics, which include a summary of the authors, study design intervention category, intervention type, study country, setting, outcome, adherence measurement, and study findings. The study design refers to the methodology and statistical methods employed in a study to collect and analyze data. In this study, the site where the intervention was conducted is referred to as the intervention setting, which could be in a hospital (hospital-based) or in the community where the patients reside (community-based). The outcome measure was adherence or correlates of adherence. The adherence measurement states how adherence was measured in each study while the study findings present the results of adherence measured. Table 2 was adapted from a systematic review [18] and summarizes the intervention categories and classifies interventions into structural, biological, behavioral, cognitive, affective, and combination (mixture of some or all the categories). 

The study designs consisted of ten cohort studies [24,25,29,33,34,37,38,47,52,53], four pre-post studies [26,28,35,36], sixteen randomized controlled trials [27,31,32,39,40,41,42,43,44,45,46,48,49,50,51,54], and only one cross-sectional study [30].

The intervention categories as described on Table 2 comprised of structural intervention [24,26,29,32,33,34,35,51], which involved intervention types such as community health worker’s home visits [24], community-based support (CBAS) groups [24,33], option B+ [25], mobile pharmacy [34], semi-mobile clinics [35], micro clinics, and community-based care by personal digital assistant [51]. Interventions that were within the affective category [24,25,26,27,28,30,31,41,42,43,44,50,54] included counseling [27,28,38,43], peer support groups [30], adherence treatment supporter [37,42], and psychotherapy [46]. Interventions in the cognitive category [39,40,49,50] involved patient education [39] and active visualization [40]. Interventions in the behavioral category [24,25,29,31,32,41,44,45,47,48,54] involved appointment diary [29], cell phone adherence sessions [41], and text messaging [45,48] while the biological category [52,53] was made of the food ration [52] and food assistance [53]. However, there were some studies that had a combination of two or more intervention types, which translated into the combination intervention category [24,26,29,31,32,41,44,50,54].

Six studies were from Kenya [24], two from Cameroon [25,45], five from Uganda [26,28,42,43,47], nine from South Africa [27,33,37,40,41,46,48,49,50], four from Nigeria [30,32,36,42], one from Swaziland [38], two from Zambia [39,53], one from Niger republic [52], and one from Zimbabwe [54]. The selected studies were either community-based [24,26,32,33,35,37,51,54] or hospital-based [25,27,28,29,30,31,34,36,38,39,40,41,42,43,44,45,46,47,48,49,50,52,53].

In this review, as shown on Table 1, twenty-four of the thirty-one included studies recounted a substantial increase in ART adherence in the intervention group when likened to the comparison group for a minimum of one outcome being measured and one time point in the course of the study [24,25,26,27,28,30,31,33,35,36,37,39,40,41,42,44,46,47,48,49,50,52,53,54]. Whereas, seven studies [26,29,32,34,38,45,51] reported no improvement in ART adherence post intervention. The interventions that resulted in significant effect were community-based adherence support [24,33], option B+ [25], mobile ART pharmacy [26], counseling [27,28,31,44], peer support and alarm device [30], micro-clinic [35], motivational interviewing [36], treatment supporter [37,42,54], group patient education [39], actual visualization [40], mobile phone call [41], mobile text messages [44,48], interpersonal psychotherapy [46], modifying clinic appointment [47], media education [50], family nutritional support and advice [52], and food assistance [53]. Nevertheless, some interventions like the clinic appointment diary coupled with training adherence staff [29], alarm device [31], semi mobile clinics [34], counseling [38,43], and mobile text messages [45] did not produce any significant effect. A combination intervention of community-based adherence support (CBAS) [51] and home visits also did not yield any significant effect on adherence in one study [32]. Table 2 presents the categories of adherence interventions [18].

### 3.2. Risk of Bias Assessment

In the hierarchy of evidence according to the risk of bias, RCTs supersede observational studies, although this could be reversed in some instances where bias is present, as the strength of evidence is limited [55]. Most of the observational studies were high-risk, this is because unlike the RCTs, they are not characterized by random sequence generation, allocation concealment, and in some cases blinding. Unclear risk of bias with respect to blinding of participants and personnel and in some cases outcome assessment was observed in some studies. The Cochrane risk of bias assessment was used [21] and presented on Table 3.

All the thirty-one intervention studies included in this review were assessed for risk of bias. The summary of the risk of bias by authors and their judgment of each risk of bias item is presented on Table 3. Items rated “low risk” were assigned a score of 1 while items rated “high risk” and “unclear risk” were assigned a score of 0 [56]. The mean score for the 31 included studies reviewed was 36; studies that scored less than 36 were termed “high risk” while studies with a total score above 36 were considered “low risk”. Thirteen studies were rated high risk [24,26,27,31,33,34,35,36,37,38,42,46,52], while the remaining eighteen were rated low risk [25,28,29,30,32,39,40,41,43,44,45,47,48,49,50,51,53,54].

## 4. Discussion

The goal of ART is lasting viral suppression to undetectable levels, and optimal adherence to ART is required to attain this. Several types of interventions have been used in sub-Saharan African countries to improve adherence to ART among HIV-positive patients. These interventions involving education and counseling, community-based adherence support, mobile devices, and food services resulted in long term or short term improvement in ART adherence. The thirty-one selected studies in this review support the drive to scale-up long-term ART success in SSA.

### 4.1. Effectiveness of Interventions

Majority of the studies in this systematic review that reported effective interventions were RCTs [27,31,39,40,41,42,43,44,46,49,50,54]. In determining the effectiveness of interventions, the RCT has proven to be the most reliable in providing evidence and has been considered the gold standard for evaluating the effectiveness of interventions over the past decade [57]. While the concept of gold standard relates to research design, a broader perspective to fully appraising the evidence of interventions as the gold standard is demonstrated in its ability to function, be implemented and serve its purpose [58]. Thus, when searching for answers to the clinical research question concerning the evaluation of diverse treatments, the RCT is primarily recommended because of its propensity to minimize bias [59]. Although a significant improvement in the intervention group denotes the intervention’s success, it is more beneficial to consider the effect size, which explains the magnitude of the effect and not just the statistical significance. Additionally, effect size is independent of sample size whereas *p* value depends on both the effect size and sample size [60]. Unfortunately, information regarding the effect size for most studies in this systematic review was not clearly stated. Subsequent interventions should base judgment of their primary findings on effect size and not solely on statistical significance.

Furthermore, in concluding on the effectiveness of an intervention, a considerable length of time may be examined in order to determine a substantial impact. In this review, one study [39] reported a reverted improvement in adherence following cross-over after three months, implying the inauthenticity of the intervention. This suggests that the short-term effect of interventions may not be generalizable as its sustenance is not guaranteed. Additionally, since ART is a life-long behavior, and optimal adherence is required for achieving maximum viral suppression, interventions with ephemeral effectiveness may just offer diminutive impact to treatment success. Thus, in order to validate the effectiveness of an intervention, prospective studies may need to observe the effectiveness of these interventions for longer duration so as to ensure lasting impact. Less than half of the included studies in this systematic review were observed for a minimum of one year [19,21,22,24,25,26,28,31,35,36,39,42], the remaining were mostly six months and below. It is suggested that more studies in sub-Saharan Africa adopt interventions with longer duration, as this may further authenticate study findings. Additionally, further studies could focus on developing a clear standard for evaluating successfulness of adherence interventions and duration of observation.

### 4.2. Adherence in Key Affected Populations 

Additionally, evident in this review was the paucity of studies on interventions relating to ART adherence among HIV key affected populations such as men who have sex with men (MSM), injection drug users (IDU), sex workers, people in prisons and other closed settings, and transgender people. In 2018, it was reported that these groups together with their sexual associates accounted for over half of the global incidence [61]. As such, it is important to conduct studies among these populations, to investigate issues peculiar to them such as linkage to care and commitment to treatment regimen, most importantly their adherence to ART, which is fundamental in managing HIV infection. Additionally, noncompliance to medications has been reported to be a characteristic behavior of these affected populations, evidenced by low adherence rates [62,63], although some studies reported over 90% adherence among MSM [64]. Many factors responsible for poor adherence in these populations include HIV stigmatization, fear of healthcare-seeking and denial of care, social isolation, poor access to health services, and psychological issues such as depression. In respect of this, further intervention studies on adherence should be considered in order to eschew the implications of nonadherence, which include transmission of resistant strains, thereby limiting the therapeutic options of newly infected patients.

### 4.3. Assessment of Adherence

In assessing adherence, it is important to discuss the different measures used by authors to arrive at their findings. From this review, self-report appeared to be the predominant measure of adherence [25,26,27,29,30,32,33,36,41,44,45,46,49,50,51,52,54] employed in SSA. Other adherence measures included pill count [24,31,33,34,42,43,46,50,51,52,53], pharmacy refill [25,29,32,37,45,47], electronic adherence monitoring device (EAMD) [48], plasma viral load [38,40], nevirapine hair concentration [47], medication possession ratio [53], and appointment scheduling [28,35,39,47].

There is no “gold standard” for measuring adherence as each of these assessments have strengths and weaknesses; however, the choice of the assessment method will greatly depend on the economic setting of the study. This is because some assessment methods are capital-intensive, and some study locations are resource-rich while others are resource-limited. These are some of the challenges associated with the choice of adherence measurement for instance, pharmacy refill and self-report are mostly employed in HIV/AIDS hospital settings while Medication Event Monitoring System (MEMS) are commonly used in clinical studies [65]. Due to its ease of use and affordability, self-report has been the most commonly used in resource-limited settings (RLS) [65]. This is consistent with the findings of the present systematic review, which revealed fifteen [25,26,27,29,30,32,36,39,41,44,45,46,49,50,54] out of thirty-one studies employed a self-report; with twelve studies reporting significant results [25,26,27,28,36,39,41,44,46,49,50,54]. It is also note-worthy to state that these twelve studies constituted half of the twenty-four studies with significant findings. Self-report is associated with many advantages, which makes it the most commonly used measure of adherence [66].

Besides its ease of use and validity that propels it to be the most widely used adherence measure, the self-report is consistent with objective methods of measuring adherence such as plasma viral load monitoring and MEMS [67]. Other advantages of a self-report in RLS include affordability and low staff requirements, it is also considered to be robust and an apt indicator of adherence [65]. The major demerit of self-report is the overestimation of adherence due to recall bias and social desirability. This mostly stems from the patient’s fear of being judged by the healthcare providers or the consequences of providing negative feedback, which compels them to give inaccurate adherence reports [68]. Despite its demerits, majority of studies in sub-Saharan Africa, especially in RLS, employ its use. We recommend that concrete justification for further use is researched.

Appointment scheduling, which is also an early warning indicator (EWI), is also considered to be subjective, although the results can be fetched from the clinic’s attendance records [65]. It is similar to the subjective self-report assessment but more objective. However, it is prone to manipulations by clinic staff [69]. Pill count and pharmacy refill on the other hand are the commonly used objective measures due to their relatively inexpensive nature and ease of use. Pharmacy refill is a validated measure of ART adherence that relates to viral load [70]. The draw-backs of pharmacy refill include pill dumping or sharing [65], the need of a closed pharmacy system, its dependence on accurate and reliable records [69], and its inability to predict or detect viral rebound in patients [71]. The disadvantage of pill count, which is also an EWI, include pill dumping and limited availability. Another drawback is that it is difficult to keep record of pharmacy visits and refills when patients obtain their medications from different pharmacies. Even though most patients in RLS return to their primary healthcare providers for free treatment and refill; this makes pharmacy refill a more feasible adherence measurement [65].

Other adherence measures seldom used as reported in this review include EAMD, viral load monitoring, hair concentration, medication possession ratio, and appointment diary. The EAMD entails recording every medication bottle opening thus providing a more reliable proof of medication-taking behavior, nonetheless this is not without demerits. In the event of a single opening, misclassification bias might occur; a situation where multiple doses could be taken out for future dosing (pocket doses) or no doses taken out at all despite opening (curiosity openings) thereby altering with its accuracy [72]. In both cases, evaluation is achieved mostly during a clinic visit or at the time of a study, which is probably long after the occurrence of the adherence gap [73]. To avoid that, real-time adherence monitoring (RTAM) devices were introduced, which are EAMD designed to deliver instant information on dosing events, this has proven to be more beneficial in monitoring adherence actively and promptly between clinic visits or in study visits [74]. RTAM devices that have proven to be feasible and reliable lately are automated medication bottles that possess lasting battery half-lives capable of containing medication supplies for a period of 30 days. It functions by transmitting a time-stamped cellular signal to a central web-based server at each opening of the device; this denotes a dosing event and is recorded [75]. Though information on adherence could be examined in real-time thus enabling prompt adherence intervention, internet connection is required for this task thereby rendering this measure less feasible especially in RLS. Other cons of this measure would be its inability to confirm medication ingestion [76], and a lack of privacy as patients may have to travel around with the device [77]. Other accurate measures although expensive, include direct methods such as measuring drug levels or its metabolite in urine or blood, detecting an added biomarker to the drug formulation, and direct observed therapy [78]. Deliberations on measuring stool and urine samples daily could be considered for further studies.

Validating the measurement of adherence against viral load is beneficial [65], and attaining undetectable viral load is also considered to be one of the most common measures of ART adherence. A high adherence level of 95% was previously associated with undetectable viral load [6] thus equating viral suppression with adherence. However, in recent times, adherence levels between 80 and 85% is sufficient for viral suppression, thereby making undetectable viral load an unsatisfactory proxy for maximum adherence. It should also be noted that viremia is evident long after the occurrence of an adherence gap [69].

In a bid to manage the inevitable limitations of the various measures of adherence, newer pharmacological measures [79] have been introduced that possess the ability to quantify medication adherence and exposure over time. The advantage of these new measures is its ability to expose both medication adherence and pharmacokinetics, which involves absorption, distribution, metabolism, and excretion in one evaluation. Dried blood spots (DBSs) and hair are the obtainable mediums that aggregate the measurement of ART adherence exposure [69].

### 4.4. Other Results

In this systematic review, interventions in the affective category and behavioral category were the most common intervention categories, while counseling and treatment supporter were the most common intervention types. The use of counselors though cumbersome has been found to be effective in improving adherence as reported in a systematic review [80]; treatment supporter intervention also yielded similar success [81,82].

Additionally, in this systematic review, more studies came from South Africa [27,33,37,40,41,46,48,49,50], Uganda [26,28,42,43,47], and Nigeria [30,32,36,44]. This finding is not surprising because the highest global disease burden of HIV lies in South Africa and Nigeria [83]. Furthermore, these countries account for about half of all new infections in sub-Saharan Africa annually [84]. This explains why more studies emanate from these countries and it is also a reason for the substantial funding of HIV research in these countries.

Furthermore, the results of this systematic review revealed that hospital-based interventions [25,27,28,29,30,31,34,36,38,39,40,41,42,43,44,45,46,47,48,49,50,52,53] were more common than community-based interventions [24,26,32,33,35,37,51,54]. This could be likened to the fact that hospital patients are more accessible in the hospitals than the community. Moreover, stigmatization is minimal in the hospital setting than the community, which makes the hospital a more preferable setting. Additionally, health personnel are mostly involved in hospital-based interventions, which is safer and more promising of authentic results than employing services from the community.

### 4.5. Limitations

The limitations of this systematic review include the unavailability of studies targeted at a key affected population such as MSM, female sex workers (FSWs) and orphans and vulnerable children, and the elderly. Some interventions as reported by some studies were at risk of bias, as study protocols were not duly followed. Additionally, because adherence is a life-long behavior, and there is no clear set standard period for observing interventions, the authors utilized the information from the studies to evaluate the successfulness of the interventions.

## 5. Conclusions

In conclusion, a wide range of studies on ART adherence interventions was done among HIV positive adults in sub-Saharan Africa. Many quality studies such as RCTs and cohorts were present; despite the high-cost and ethical limitations of RCTs. Additionally, various types of interventions were used in both hospital and community settings in different countries to improve adherence; although the majority proved effective in both settings, some failed to show any effect. In addition, among the various methods of assessing adherence, subjective self-report though unreliable, proved to be the commonly used measure of adherence. It is recommended that objective methods of assessment that are more reliable be used in future studies. Lastly, further studies should focus on closing significant evidence gaps on interventions for improving adherence. These gaps include effectiveness in key affected populations, long-term effectiveness, and quality studies. 

## Figures and Tables

**Table 1:** Summary of characteristics of interventions on ART adherence in sub-Saharan Africa.

Author	Study Design	Intervention Category	Intervention Type	Study Country	Intervention Setting	Outcome	Adherence Measurement	Follow-Up Duration	Findings
Achieng et al. (2012)	Prospective cohort	Structural	Community health worker’s home visits, pharmacy counseling, community-based support groups, and unannounced pill counts by clinicians.	Kenya	Community-based	Time to treatment failure as defined by a detectable HIV-1 viral load	Pill count	1 year	Time to treatment failure was longer in support groups. Better adherence and improved pill counts in support groups.
Atanga et al. (2018)	Prospective cohort	Structural	Option B+	Cameroon	Hospital-based (outpatient)	Adherence	(1) Pharmacy refill (2) Self-report	1 year	Improvement in adherence was seen in the intervention group from 6 months to 12 months with 92.7% viral suppression. Low adherence was associated with treatment failure.
Bajunirwe et al. (2019)	Pre-post study	Structural, affective	Mobile ART pharmacy and counseling	Uganda	Community-based	Adherence, waiting time, viral suppression	Self-report	1 year	No improvement in waiting time. Number of missed doses significantly declined 12 months post-intervention. Proportion of detectable viral load in patients decreased post-intervention.
Bhana et al. (2014)	Pilot randomized controlled trial	Affective	Counseling by lay counselors to pre-adolescents and their families (VUKA) vs. SOC	South Africa	Hospital-based (outpatient)	Adherence, treatment knowledge, care-giver communication, illness stigma	Self-report	3 months	Greater improvements in ART adherence in VUKA post-intervention.
Boeke et al. (2018)	Pre-post study	Affective	Lay workers counseling	Uganda	Hospital-based (outpatient)	Adherence, linkage to care, retention	Appointment scheduling	1 year 6 months	Adherence of patients to appointment schedules was improved.
Boruett et al. (2013)	Quasi-experimental, (cohort)	Behavioral	Clinic appointment diary, modifying self-report adherence questions, staff training, visiting support facilities and use of monitoring data vs. SOC	Kenya	Hospital-based	Adherence to medication and clinic appointment,	(1) Pharmacy refill (2) Self-report	11 months	There was maximum adherence (100%) in both groups and at baseline and pot-intervention. No change was observed.
Chime et al. (2018)	Cross-sectional	Affective	Peer Support groups vs. standard of care	Nigeria	Hospital-based (outpatient)	Adherence	Self-report	No post-intervention	Better adherence was seen in the intervention group compared to the control group.
Chung (2011)	Randomized controlled trial	Affective, behavioral	Counseling vs. alarm device vs. counseling + alarm, vs. SOC	Kenya	Hospital-based	Adherence, Viral load, CD4 count, mortality,	Pill count	1 year 6 months	Adherence was significantly improved and treatment failure decreased post-intervention (18 months follow-up) whereas no significant impact on adherence and viral failure was observed for alarm use.
Coker et al. (2015)	Three-arm Randomized controlled trial	Behavioral, structural (combination)	Alarm daily reminder + follow up calls from peer educators + adherence support (CBAS) Vs. CBAS + home-based treatment partner + SOC	Nigeria	Community-based	Viral suppression	(1) Self-report (2) Pharmacy refill	9 months	There was no significant change in viral suppression between both (intervention and control) groups post-intervention.
Fatti et al. (2012)	Observational multicohort	Structural	Community-based adherence support (CBAS)	South Africa	Community-based	Viral suppression, Patient retention, and mortality rate	(1) Self-report (2) Pill count	5 years	There was significant difference in viral suppression between intervention and control groups 6 months post-intervention.
Gorman et al. (2015)	Retrospective cohort	Structural	Semi-mobile clinics	Kenya	Hospital-based (outpatient)	Adherence, CD4 count, mortality, HIV treatment retention	Pill count	5 years	There was no change in adherence and CD4 count between intervention group and control group
Hickey et al. (2015)	Quasi experimental study (pre-post)	Structural	Microclinics vs. SOC	Kenya	Community-based	Linkage to care and ART concentration in hair	(1) Drug levels (Nevirapine concentration in hair) (2) Appointment scheduling	6 months	The intervention group had less NVP hair concentration than control group. Microclinic could possibly improve ART adherence.
Holstad et al. (2012)	Quasi-experimental, two group post-test only design	Affective, Cognitive	Motivational interviewing (MI) vs. Health promotion program (HPP)	Nigeria	Hospital-based (outpatient)	Adherence, knowledge of HIV, condom use, safe sex	(1) Self-report	6 months	Higher mean adherence in MI group compared to HPP post-intervention.
Igumbor et al. (2011)	Retrospective cohort	Affective	Adherence Treatment supporter (patient advocate)	South Africa	Community-based	Virologic outcome	(1) Pharmacy refill	6 months	There was improved viral suppression in intervention group (<400 copies/mL) at 6 months; improved drug pickup rate of >95% and increased retention in care.
Jobanputra et al. (2015)	Retrospective cohort	Affective	Enhanced adherence counseling by lay counselors vs. SOC	Swaziland	Hospital-based (outpatient)	Viral suppression, CD4 count	Plasma viral load	6 moths	No change in odds of viral re-suppression between EAC group and SOC post-intervention.
Jones et al. (2013)	Randomized controlled trial	Cognitive	Group patient education vs. individual patient education	Zambia	Hospital-based (outpatient)	Adherence to medication and clinic visits	(1) Self-report (2) Appointment scheduling	6 months	Participants of group intervention had enhanced adherence, but following crossover, gains were not sustained to the individual intervention.
Jones et al. (2018)	Randomized controlled trial (RCT)	Cognitive	Active visualization	South Africa	Hospital-based (outpatient)	Adherence measured by viral load	Plasma viral load	2 months	There was change in viral load scores and higher suppression in intervention group
Kalichman et al. (2018)	Randomized controlled trial (RCT)	Behavioral	Mobile phone counseling vs. a contact matched control	South Africa	Hospital-based (outpatient)	Adherence	Self-report using VAS	2 weeks	Intervention group significantly improved in ART adherence post-intervention
Kunutsor et al. (2011)	Two-arm Randomized controlled trial	Affective	Treatment supporter (TS) vs. SOC	Uganda	Hospital-outpatient (Rural)	Adherence and clinic attendance for refills	Pill count	7 months	TS participants had greater optimal adherence.
Kiweewa et al. (2013)	Randomized controlled trial	Affective	Peer support counseling	Uganda	Hospital-based (outpatient)	Adherence, virologic suppression	Pill count	1 year	No change in adherence and viral suppression between intervention and control groups.
Maduka and Tobin-West (2013)	Randomized Controlled Trial	Affective, behavioral	Adherence counseling, mobile-phone text messages, Standard of Care	Nigeria	Hospital-based (Urban)	Adherence, Immunological outcome	Self-report	4 months	The intervention group had higher adherence and CD4 count than control group post-intervention.
Mbuagbaw et al. (2012)	Randomized controlled trial	Behavioral	Text messages vs. standard of care	Cameroon	Hospital-based (outpatient)	Adherence	(1) Pharmacy refill (2) Self-report	6 months	No significant effect was seen between groups post-intervention.
Moosa and Jeenah (2012)	Prospective Randomized Controlled Trial	Affective,	Interpersonal psychotherapy vs. pharmacotherapy	South Africa	Hospital-based	Adherence	(1) Self-report (2) Pill count	6 months	Adherence improved greater in the intervention group compared to the control group
Obua et al. (2014)	Cohort	Behavioral	Appointment system, fast-tracking, longer prescription	Uganda	Hospital-based-outpatient	Adherence	(1) Pharmacy refill (2) Appointment scheduling using appointment diary	1 year	Reduced missed appointments improved adherence
Orrell et al. (2015)	Randomized Controlled Trial	Behavioral	Text messages vs. standard of care	South Africa	Hospital-outpatient	Adherence, viral load treatment interruption count	Electronic adherence monitoring device (EAMD)	1 year	Although not statistically significant, mean ART adherence increased more in intervention group. However, viral suppression was more in control group.
Peltzer et al. (2012)	Two-armed Randomized Controlled Trial (RCT)	Cognitive	Medication Adherence Training + structured three session group intervention vs. Standard of Care (SOC)	South Africa	Hospital-bases (outpatient)	Adherence, Immunologic outcome, depression level	Self-report	3 months	Intervention group had more increase in ART adherence and CD4 count.
Robbins et al. (2015)	Randomized controlled trial	Combination (cognitive and affective)	Media education (masivukeni) vs. standard of care counseling	South Africa	Hospital-based (outpatient)	Adherence,	(1) Self-report (2) Pill count	6 weeks	Intervention group experienced more increase in adherence while control group decreased.
Selke et al. (2012)	Prospective cluster randomized controlled clinical trial	Structural	Community-based care by personal digital assistant vs. SOC	Kenya	Community-based	Adherence, Viral load, CD4 count,	(1) Self-report (2) Pill count	1 year	No statistical significance between intervention and control arms at 6 months and 12 months
Serrano et al. (2010)	Retrospective cohort	Biological	Family nutritional support + nutritional advice vs. SOC	Niger	Hospital-based (outpatient)	Adherence	(1) Self-report interviews (2) Pill count	6 months	Increased mean adherence post-intervention and improved CD4 count.
Tirivayi et al. (2012)	Retrospective cohort	Biological vs. SOC	Food ration (Food assistance)	Zambia	Hospital-based	Adherence, weight gain, CD4 count	Pill count (medication possession ratio)	6 months	Intervention arm had higher ART adherence however, there was no change in CD4 count and weight.
Willis et al. (2019)	Randomized controlled trial (RCT)	Affective, behavioral	Community adolescent treatment supporter (CATS), pill boxes, monthly support group vs. SOC	Zimbabwe	Community-based	Adherence, psychological wellbeing, quality of life	Self-report	1 year	The intervention arm had more likelihood of adhering to ART than the control arm.

**Table 2:** Categories of adherence interventions.

Categories	Description	Examples
Affective	Using emotional support to affect ART adherence	Peer support (social support) Treatment with antidepressants counseling
Behavioral	Using direct behavior modification to affect ART adherence	Reminder devices (like pill boxes, alarms, mobile-phone text messages, pager messages)Cash incentives Directly Observed Therapy (DOT)
Biological	Using improved physical ability to take ART to affect ART adherence	Vitamin/micronutrient supplements Food rations/assistance
Cognitive	Using teaching, clarification or instruction to affect ART adherence	Patient education Media education materials (such as audio, video, or reading materials)
Combination	Using a combination of one more intervention categories to affect ART adherence	Peer support, Patient education Food rations
Structural	Using changes in the delivery structure or additional service structures to affect ART adherence	ART delivery in community centers Income-generating activities for ART patients Community mobilization

**Table 3:** Risk of bias ratings for each study included.

	Item ^1^	Item ^2^	Item ^3^	Item ^4^	Item ^5^	Item ^6^	Item ^7^	Total *n* (%)	Bias
Achieng et al. (2012)	0	0	0	0	1	1	0	2 (29%)	High
Atanga et al. (2018)	0	0	0	0	1	1	1	3 (43%)	Low
Bajunirwe et al. (2019)	0	0	0	0	1	1	0	2 (29%)	High
Bhana et al. (2014)	1	0	0	0	0	0	0	1 (14%)	High
Boeke et al. (2018)	0	0	0	0	1	1	1	3 (43%)	Low
Boruett et al. (2013)	0	0	0	0	1	1	1	3 (43%)	Low
Chime et al. (2018)	0	0	0	0	1	1	1	3 (43%)	Low
Chung (2011)	1	1	0	0	0	0	0	2 (29%)	High
Coker et al. (2015)	1	1	1	1	1	1	0	5 (71%)	Low
Fatti et al. (2012)	0	0	0	0	1	1	0	2 (29%)	High
Gorman et al. (2015)	0	0	0	0	1	1	0	2 (29%)	High
Hickey et al. (2015)	0	0	0	0	1	1	0	2 (29%)	High
Holstad et al. (2012)	0	0	0	0	1	1	0	2 (29%)	High
Igumbor et al. (2011)	0	0	0	0	1	1	0	2 (29%)	High
Jobanputra et al. (2015)	0	0	0	0	1	1	0	2 (29%)	High
Jones et al. (2013)	1	0	0	1	1	1	0	4 (57%)	Low
Jones et al. (2018)	1	1	0	0	1	1	0	4 (57%)	Low
Kalichman et al. (2018)	1	0	0	1	1	1	1	5 (71%)	Low
Kunutsor et al. (2011)	1	0	0	0	0	0	0	1 (14%)	High
Kiweewa et al. (2013)	1	1	0	0	1	1	0	4 (57%)	Low
Maduka and Tobin-West (2013)	1	1	0	1	1	1	1	6 (86%)	Low
Mbuagbaw et al. (2012)	1	1	1	0	1	1	1	6 (86%)	Low
Moosa and Jeenah (2012)	0	0	0	0	0	1	0	1 (14%)	High
Obua et al. (2014)	0	0	0	0	1	1	1	3 (43%)	Low
Orrell et al. (2015)	1	1	0	0	1	1	1	5 (71%)	Low
Peltzer et al. (2012)	1	0	0	0	1	1	0	3 (43%)	Low
Robbins et al. (2015)	1	0	0	0	1	1	0	3 (43%)	Low
Selke et al. (2012)	1	1	0	0	1	1	0	4 (57%)	Low
Serrano et al. (2010)	0	0	0	0	1	1	0	2 (29%)	High
Tirivayi et al. (2012)	0	0	0	0	1	1	1	3 (43%)	Low
Willis et al. (2019)	1	0	0	0	1	1	0	3 (43%)	Low

Note. ^1^ Random sequence generation, ^2^ allocation concealment, ^3^ Blinding of participants and personnel, ^4^ Blinding of outcome assessment, ^5^ Incomplete outcome data, ^6^ Selective reporting, ^7^ Other bias. “H” = 0, “U” = 0, “L” = 1. Mean score = 36. Higher scores and percentages denote lower risk of bias.

## Data Availability

Data sharing not applicable.
